# Transketolase Serves as a Biomarker for Poor Prognosis in Human Lung Adenocarcinoma

**DOI:** 10.7150/jca.69583

**Published:** 2022-05-13

**Authors:** Cong Niu, Wenjia Qiu, Xiangyang Li, Hongqing Li, Ji'an Zhou, Huili Zhu

**Affiliations:** Department of Respiratory Medicine, Huadong Hospital, Fudan University, Shanghai, China

**Keywords:** Transketolase, lung adenocarcinoma, prognosis, biomarker

## Abstract

Despite apparently having completed surgical resection, approximately half of resected early-stage lung cancer patients relapse and die of their disease. Adjuvant chemotherapy reduces this risk by only 5% to 8%. Thus, there is a need for better identifying the drivers of relapse, who benefits from adjuvant therapy, and novel targets in this setting. Although emerging evidence has suggested a strong link between the pentose phosphate pathway (PPP) and cancer, the role of transketolase (TKT), an enzyme in the nonoxidative branch of the PPP that connects PPP and glycolysis, remains obscure in Lung adenocarcinoma (LUAD). In this study, TKT expression was first identified in The Cancer Genome Atlas (TCGA) and then validated with our database. TKT was upregulated at protein levels in cancer compared with normal tissues (P <0.05), and high TKT expression was associated with advanced tumor stage in our cohorts. Besides, TKT inhibitor promotes tumor cell apoptosis and cell cycle blockade. Clearly, TKT plays a critical role in LUAD progression and prognosis and could be a potential biomarker for prediction of recurrence after lung cancer resection.

## Introduction

Lung cancer is currently the most common malignancy and the leading cause of cancer-related deaths in the word. Despite recent significant medical advances, the disease remains the leading cause of cancer death and protends one of the poorest 5-year survival rates among all cancer types 1. Lung adenocarcinoma (LUAD) is the most common histologic type of lung cancer, accounting for over 40% of total lung cancer cases 2, 3. The high mortality rate of LUAD is partially due to the poor diagnostic rate in the early stage, high rates of recurrence, and difficulty for therapy 4, 5. A better understanding of the underlying molecular mechanisms of progression would significantly benefit the clinical outcome. Besides, it is certainly of great interest to identify novel biomarkers and therapeutic targets for LUAD.

Rewiring of metabolism has been highlighted as a hallmark of cancer since nearly a century ago by Otto Warburg 6. Cancer metabolic reprogramming, namely the Warburg effect, which primarily on anaerobic glycolysis to generate adenosine 5'-triphosphate (ATP) instead of mitochondrial oxidative phosphorylation, even in the presence of oxygen, with increased lactate and ATP production and increased glucose uptake 7, 8. This metabolic shift plays an important role in tumor immune escape, progression and resistance to immune-, radiation- and chemo-therapy 8, 9. The pentose phosphate pathway (PPP), a branch of glycolysis, can work in parallel to glycolysis in glucose degradation in most living cells. Different studies demonstrated PPP was a vital factor in cancer growth and survival, maintaining reactive homeostasis against oxidative stress during metastasis progression 10-13.

Transketolase (TKT) is the main enzyme involved in non-oxidative PPP, which involves three human genes: transketolase (TKT) and two TKT-like genes (TKTL1 and TKTL2) 14. Several studies had been performed to determine the associations between the TKT genes family and cancer carcinogenesis 15-18. The expression of TKT in breast cancer was elevated, companied with the poor prognosis 19. Recently, it has been demonstrated that overexpression and higher enzymatic activity of transketolase increase cisplatin resistance, and their silencing or combined treatment with cisplatin could restore cisplatin sensitivity 9. In colorectal cancer tissues, TKTL1 was significantly upregulated, and correlated with liver metastases and poor disease-free survival 20. The prognosis of ovarian cancer patients with high expression of TKTL2 is worse than that of patients with low expression of TKTL2 21. Therefore, TKT and TKTL1/2 may serve as a potential biomarker to predict tumor prognosis. Nevertheless, the potential clinical value of TKT and TKTL1/2, especially in terms of prognosis and development of LUAD, had not been fully elucidated.

In this study, we reveal the clinical significance of TKT in the progression and metastasis of LUAD. TKT plays an important regulatory role in the dynamic switch of glucose metabolism. Combined therapy based on the novel target TKT could be an improved treatment for LUAD.

## Materials and Methods

### Clinical samples

Patients who were histologically confirmed as having lung adenocarcinoma by lung resection according to the classification criteria of the International Association for Lung Cancer Research-8 (IASLC) 22 were collected from Huadong Hospital from September 2012 to December 2013. A total of 161 Chinese patients, aged 18-80 years old, with good major organ function and normal clotting function were selected. The detailed inclusion criteria were described as follows: (1) Radical surgical resection was performed. (2) Postoperative pathology confirmed as primary lung adenocarcinoma. (3) Complete clinicopathological data and follow-up data; (4) Preoperative chemotherapy, radiotherapy, immunotherapy and targeted therapy were not performed. Exclusion criteria were stated: (1) Complications such as serious cardiovascular and cerebrovascular diseases or diabetes. (2) Pathological types were confirmed by immunohistochemistry to be mixed. Surgical tumor samples were immobilized in formalin for more than 24 hours and paraffin-embedded tissues were pending for immunohistochemistry and fluorescence experiments. The studies involving human participants were reviewed and approved by the Ethics Committee of Huadong Hospital. All study participants completed an informed consent form in accordance with the Declaration of Helsinki.

### Analysis of the Cancer Genome Atlas (TCGA)

RNA-Seq datasets containing 11093 tissues from tumor patients were downloaded from The Cancer Genome Atlas (TCGA), containing data from 730 normal tissues and 10363 all cancer tissues. After pretreatment RNA-seq data of 59 pairs of normal and Lung adenocarcinoma tissues were selected as the test set according to the patient code, ensuring that each pair of issues came from the same patient and forming a paired analysis.

### Immunohistochemistry

The tissues were cut into 4-µm-thick sections, fixed on slides, and dried for 12-24 h at 37°C. Sections were subsequently deparaffinized in xylene and rehydrated through graded ethanol and distilled water. After antigen retrieval, sections were incubated with anti-human TKT TKTL1 and TKTL2 antibody overnight at 4°C. The staining sections were incubated with the secondary antibody, then DAB chromogenic reagent was added and the slides were mounted for observation after dehydration.

### Evaluation of immunostaining

The level of TKT/TKTL1/TKTL2 expression was performed by semiquantitative analysis as described previously 23. The intensity of immunostaining was evaluated by the degree of color and then was scored as none (0), yellow (1), brown and yellow (2), and tan (3). The proportion of positive tumor cells was defined as follows: 0, 0%; 1, 1%-20%; 2, 21%-40%; 3, 41%-60%; 4, 61%-80%; and 5, 81%-100%. Both were evaluated by two independent observers who were unaware of the clinicopathologic features of the patients. The expressions of protein in all samples were finally scored by multiplying the intensity and the percentage ranging from 0 to 15. The immunohistochemistry (IHC) scores of TKT expression in NSCLC tissues were divided into two levels: low (0-3) and high (4-15).

### Western blot

The total cellular proteins were extracted with RIPA buffer (Beyotime). Equal amounts of protein (30 µg/lane) were separated on sodium dodecyl sulfate (SDS)-PAGE and then transferred to polyvinylidene fluoride (PVDF) membranes (Millipore, MA). After blocking with PBS buffer (Beyotime) containing 5% non-fat milk and 0.1% Tween 20 (Beyotime), membranes were incubated with primary antibody overnight at 4°C. Subsequently HRP-conjugated secondary antibodies were incubated for 1 hour at room temperature and developed with enhanced chemiluminescence (Beyotime).

### Immunofluorescence

Briefly, the sections were washed with phosphate buffered saline (PBS, Hyclone) at room temperature, China), then were blocked with 10% goat serum at room temperature for 60 mins and incubated with Transketolase antibody (abacm) overnight at 4°C. Slices were incubated with fluorescent secondary antibody mixture for 1h followed by PBS washing. DAPI (Beyotime) was used for nuclear staining for 5 mins After washing, the sections were covered with DAPI (Beyotime) and cover glass. Confocal laser scanning microscope (LEICA TCS SP8) was used for detection.

### Cell Proliferation assays

Proliferation assays were performed using the cell proliferation reagents WST-8 (DOJINDO, JAPAN). Briefly, A549 Cells were seeded at 2×10^4^ cells/well in a 96-well plate with DMEM containing 10%FBS for 24 h and then treated with different concentrations of oxythiamine (0-100µM) for 6,12,24 and 48 h. 10µl of WST-8 reagents were added into wells and incubated for an hour, then checked at 0, 24, 48, and 72 hours. The absorbance was recorded at 450 nm with 630 nm of reference wavelength by a scanning multiwall spectrophotometer (TECAN Spectra Fluor Plus (TECAN Austria GmbH, Austria)). Each treated group contained 6 repeated wells, and the experiment was repeated on three occasions.

### Cell cycle analysis

A549 cells were treated with different concentrations of Thiamine (10µM) or Oxythiamine (0-100µM) for 24 h and 48 h. All the cells were trypsinized, harvested, washed twice with PBS, fixed in 70% ethanol at 1×10^6^ cells/ml. Then were washed in cool PBS twice and suspended in DNA staining solution for 15 min at room temperature before flow cytometry.

### Apoptosis assay

Cell apoptosis was measured using an Annexin V FITC/PI Apoptosis Detection Kit (BD Biosciences). Briefly, A549 cells were incubated with 5 ml Annexin V-FITC and 10 ml PI in a binding buffer for 30 min at room temperature, and resuspended in the same buffer. Established cells above were tested by flow cytometry (BD FACS Aria II). Flow jo V10 were used to analyze cell apoptosis and cell cycle, respectively. This assay was performed in triplicate.

### Statistical analysis

All TCGA statistical analyses were conducted by ggplot2 package and other R packages in R software (Version 3.6.3). The results of Western blot were quantified by Image J. IHC score of TKT expression in LUAD tissues and adjacent normal tissues were analyzed by Wilcoxon rank-sum text. The impact of TKT on DFS or OS in lung adenocarcinoma patients was evaluated by univariate and multivariate Cox proportional hazards model and expressed as the hazard ratio (HR) and its 95% confidence interval (CI) after adjusting age, sex, TNM stage. Binary logistic regression models were used to evaluate the independent prognostic factors. Survival curves were constructed using the Kaplan-Meier method, and the differences between the survival curves were examined by the log-rank test.

All values were expressed as mean ± SEM. Data were analyzed by one-way analysis of variance (ANOVA) followed by Tukey's multiple comparison. Statistical analyses and graphs were performed by SPSS ver. 22.0 software (IBM) and GraphPad Prism ver. 800 (San Diego, CA). P<0.05 was considered as statistically significant differences (*P<0.05; **P<0.01; ***P<0.001).

## Results

### Expression patterns of TKT in LUAD predicted by bioinformatics

**Figure 1:**
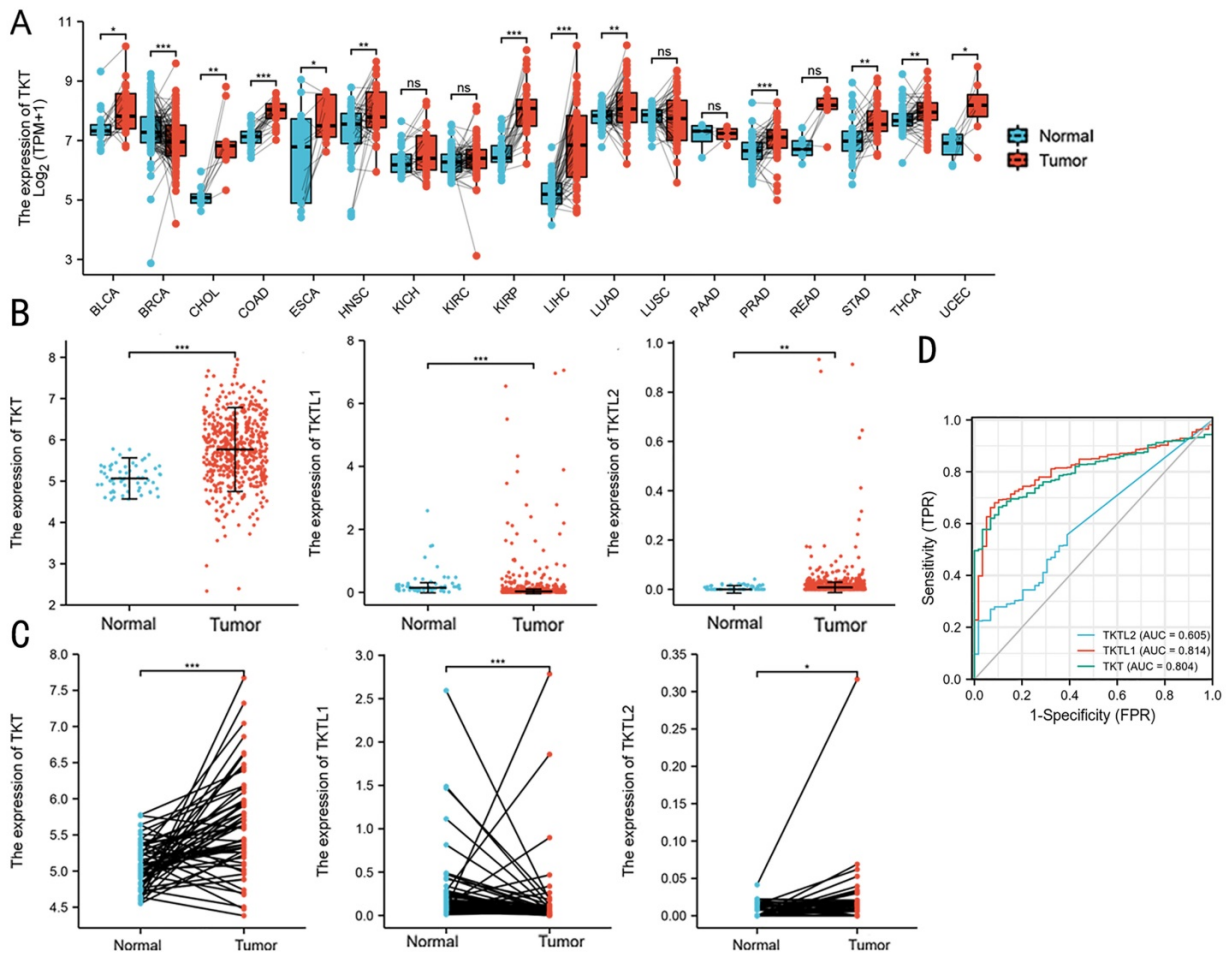
** Expression patterns of TKT in LUAD predicted by bioinformatics.** (A) Expression of TKT mRNA in TCGA database in various tumors; (B) Comparison of the expression profile of TKT and TKTL1/TKTL2 in TCGA database; (C) The mRNA was dramatically different in cancer than in normal lung tissues in 59 paired patients; (D) Receiver operating characteristic (ROC) curve analysis for the prognostic score model. The diagnostic value of TKT/TKTL1/TKTL2 mRNA in LUAD patients. *, P<0.05; **, P<0.01; ***, P<0.001.

The TCGA database was used to compare the mRNA levels of TKT and TLTL1/2 between LUAD and normal tissues. It showed that mRNA levels of TKT were up-regulated in breast, rectal, gastric and lung cancer patients (Figure 1A). Expression of TKT and TKTL2 were significantly increased in patients with LUAD, with fold change of 5.771 (P<0.001) and 0.025 (P<0.01) (Fig. 1B). Further analysis showed that TKTL1 was expressed at very low in LUAD patients in TCGA database, with fold change of 0.197 (P<0.001). Similar results were found in 59 paired tissues. The expression of TKT (P < 0.001; Fig. 1C) and TKTL2 (P < 0.05; Fig. 1C) mRNA were dramatically higher in cancer than the normal tissues. In addition, the ROC curve analyses demonstrated that TKT is a hallmark for lung adenocarcinomas that discriminates lung cancers from non-malignant lung tissues with excellent area (0.804) under curve (AUC) scores (p<0.001, Fig. 1D).

### TKT was upregulated in LUAD tissues

**Figure 2:**
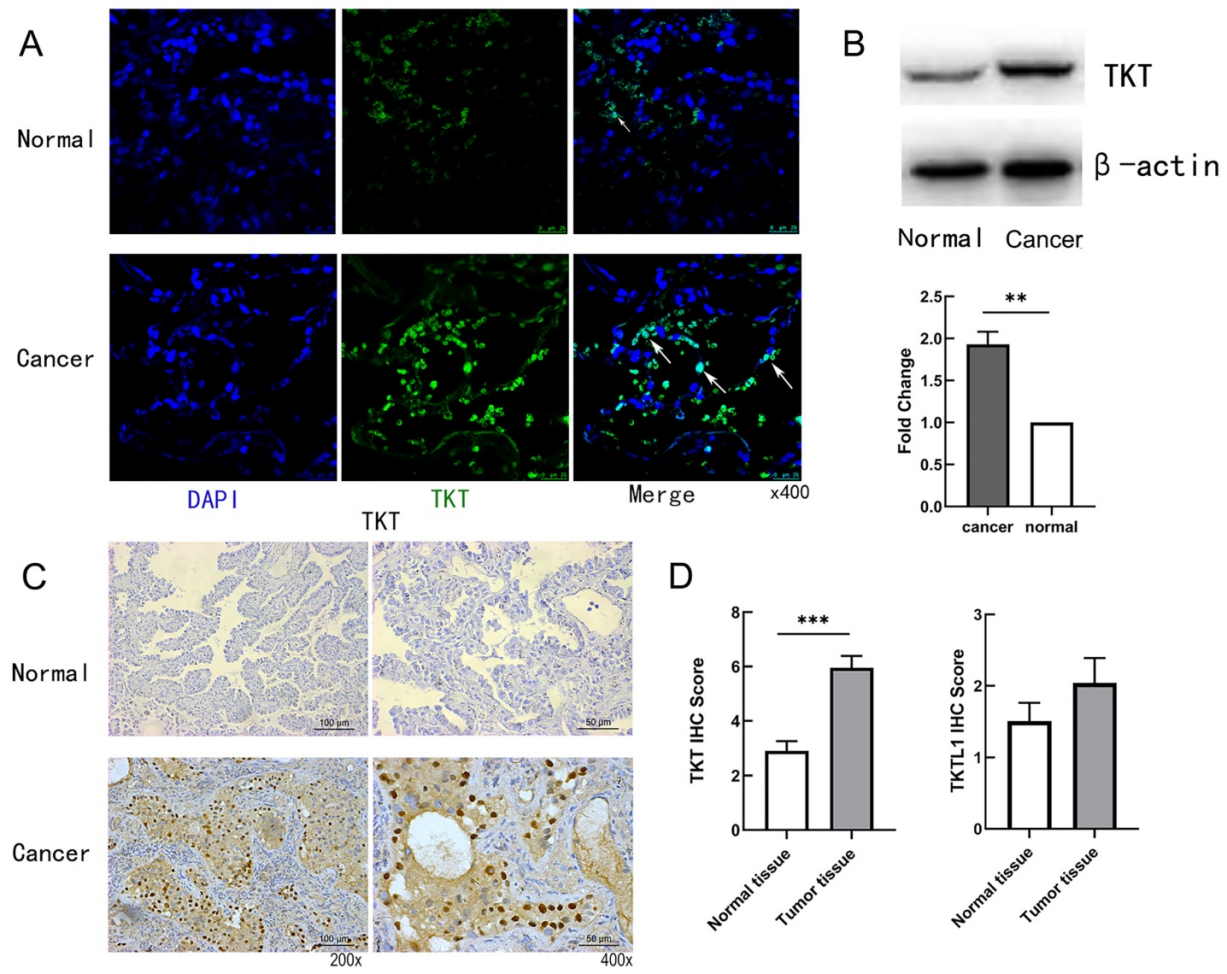
** Representative images of TKT expression in LUAD tissues and their normal controls.** (A) Representative fluorescence images of TKT identified in tumor samples from LUAD patient; (B) The protein expression level of TKT; (C) Immunohistochemical staining of TKT in LUAD samples. Original magnifications ×200 and ×400 (lower panels); (D) Expression of TKT and TKTL1 in LUAD tissues and paired adjacentnormal lung tissues by immunohistochemistry. Data are represented as the means ± S.D, and significant differences are indicated as *P < 0.05, **P < 0.01, and ***P < 0.001.

Immunofluorescence staining was used to testify the cellular location of TKT. As shown in Fig.2A, the expression of TKT was strongly positive in the tumor tissues, and the number of cells expressing TKT was significantly increased. Consistently, TKT was found to be increased in lung tumor tissues in comparison with normal tissues (Fig. 2B). TKT was mainly located in the nucleus and cytoplasm (Fig. 2C), and IHC score showed TKT in carcinoma tissues was higher than in adjacent normal tissues (p < 0.001) both in LUAD. Interestingly, we also compared the expression of TKTL1 and TKTL2. The results showed that there was no difference in TKTL1 but remain the elevated tendency (Fig. 2D).

### TKT upregulation was associated with poor prognosis in LUAD patients

**Figure 3:**
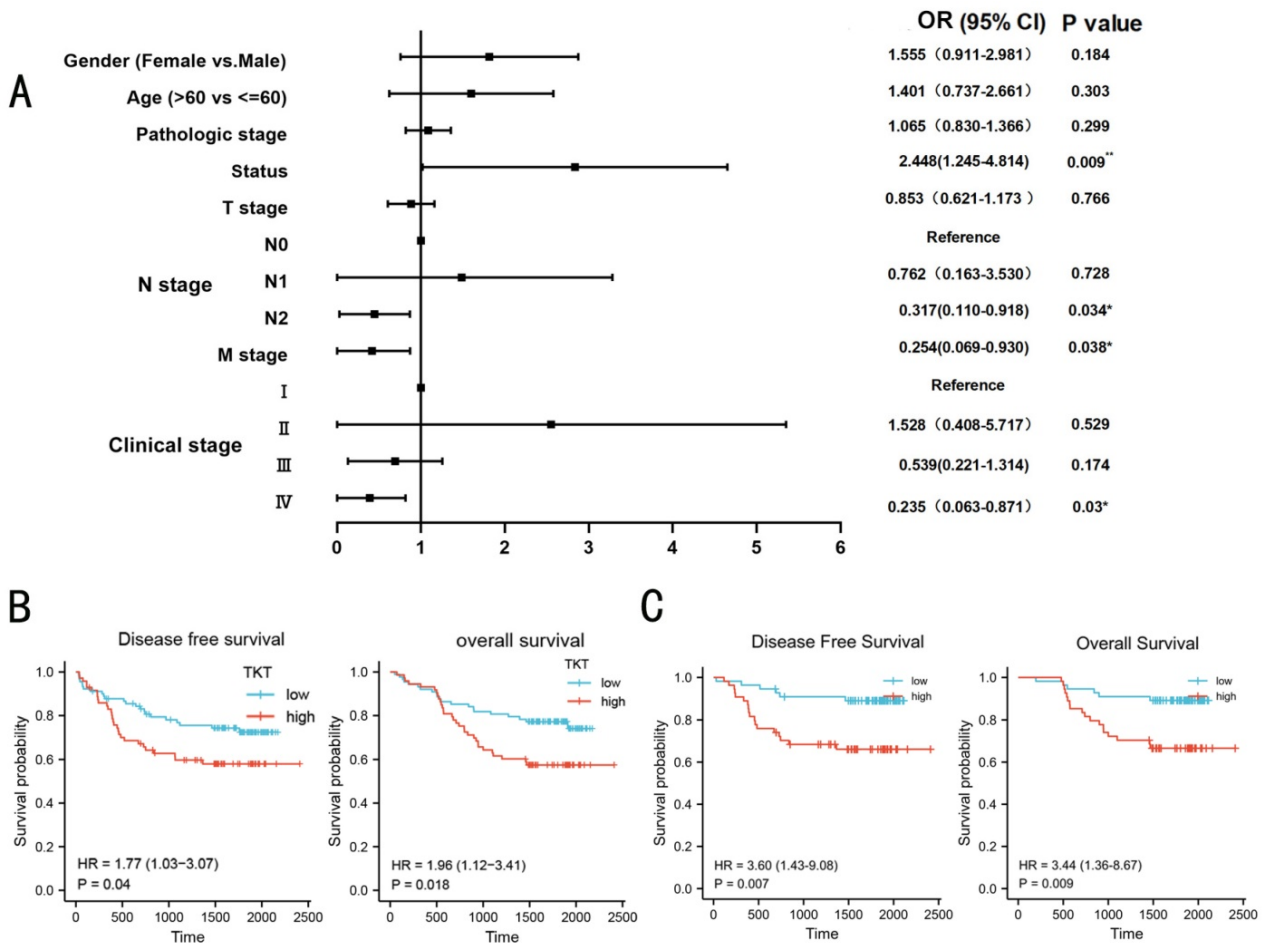
** Overexpression of TKT predicted poorer prognosis in the validation cohort.** (A) Association between TKT expression and clinicopathologic features in the LUAD validation cohort; (B) Kaplan-Meier curves for LUAD patients. The relationship of TKT with DFS and OS in LUAD patients; (C) Prognostic value of TKT expression for DFS and OS of patients with stage I LUAD patients based on the Kaplan‑Meier plotter. *, P<0.05; **, P<0.01; ***, P<0.001.

Based on TCGA datasets, the expression of TKT was used as a biomarker to predict patient survival in LUAD. We surmised that TKT had higher clinical value in LUAD and verified in our cohort. We analyzed the correlation between the TKT expression and both overall survival (OS) and disease-free survival (DFS) of LUAD patients. High TKT expression was significantly correlated with characteristics of a poor prognosis, such as shorter overall survival (P = 0.009), metastasis of tumor (P = 0.038) and more serious clinical conditions (P = 0.03) (Fig. 3A). In the multivariate logistic regression model, three independent risk factors for TKT were further screened out, including gender (OR=3.327, 95% CI 1.456-7.606, P=0.004), M stage (OR=0.081, 95% CI 0.018-0.366, P=0.001), survival status (OR=6.150, 95% CI 2.502-15.119, P=0.001). The remaining factors did not show significant statistics. The expression of TKT was also demonstrated that remain an important prognostic factor for recurrence and survival in stage I patients (Figure S1). Kaplan-Meier survival analysis showed that high TKT expression at both mRNA and protein levels was significantly correlated with shorter OS (HR, 1.96; p=0.018) and DFS (HR, 1.77; p=0.04) time of the patients (Fig. 3B). Of note, the group with lower TKT expression had better OS (HR, 3.44; p=0.009) and DFS (HR, 3.60; p=0.007) than the group with higher TKT expression in stage I patients (Fig. 3C). These results suggest that TKT may contribute to LUAD progression and metastasis.

To stringently control for any confounding effect by stage, we performed stage-stratified Cox regression for all the association analyses. TNM stage, pathological stage and TKT expression were significant negative prognostic factors for OS in univariate analysis. In multivariate Cox regression analysis TKT remained an independent negative prognostic factor (P <0.001) (Table 1). Of note, the analyses of stage I patients had similar results. Clinical factors predictive of early recurrence after surgery were gender (HR=0.395, P=0.006), age (HR=2.356, P=0.027) and T-stage (HR=0.380, P=0.008) (Table S1). Multivariate analysis indicated that gender, age and T-stage were no longer significant prognostic factors with respect to Disease Free survival while TKT high expression (HR=3.177, p=0.008) remained significant.

### TKT promoted NSCLC cells proliferation and inhibited apoptosis in vitro

**Figure 4:**
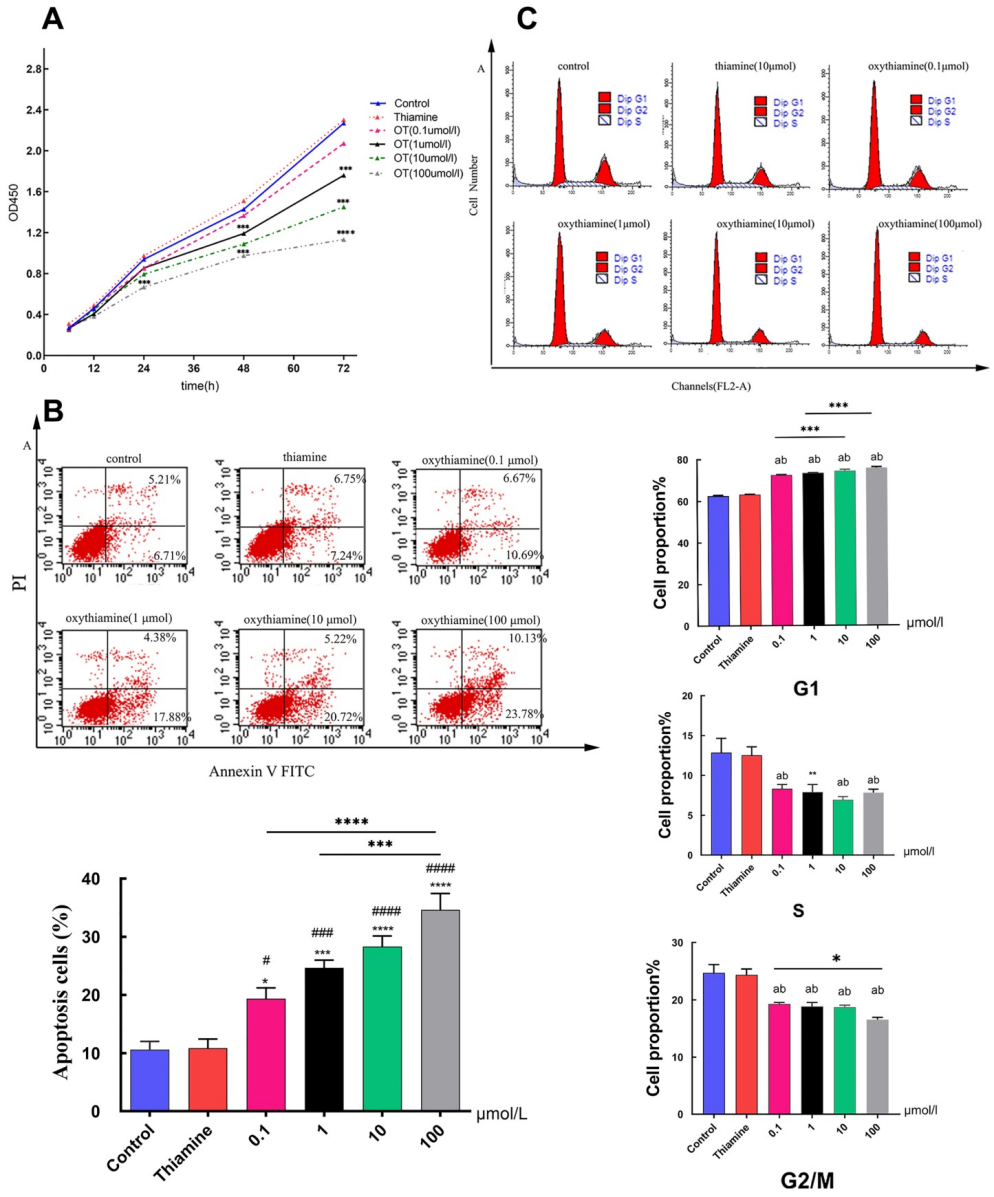
** Oxythiamine(OT) suppressed tumor cell proliferation and promoted apoptosis via inhibition of TKT.** (A) Effects of OT on the proliferation of A549 cells; (B) Effects of OT on cell apoptosis in A549 cells. The percentage of apoptotic A549 cells which treated with OT (0.1- 100μM) were 19.352±4.522%, 24.655±3.236%, 28.290±4.494% and 34.638±6.877%, respectively; (C) Effects of OT on cell cycle in A549 cells. OT induced cell cycle arrest at G1 phase. All data from three separate experiments are presented as mean ± SD. * p < 0.05; ** p < 0.01; *** p < 0.001.

Thiamine is one of the most important vitamins needed for proper cell metabolism 24. And several antimetabolites of thiamine such as metronidazole 25, pyrithiamine 26 or oxythiamine (OT) 27 have been synthesized and tested as antibiotics or cytostatic. In this study, we used thiamine and OT to investigate the effect of TKT on cell proliferation and apoptosis. The growth curve of A549 cells was obviously suppressed under the action of different concentrations of inhibitors (p < 0.001) in a time-and dose-dependent manner (Fig. 4A). We found that the percentage of apoptotic cells did not change in the thiamine group compared to the control group. However, the proportion of apoptotic cells, both early and late apoptotic, increased significantly with the use of inhibitors (Fig. 4B). We further assessed the effect of TKT on cell cycle. OT significantly induced G0/G1 arrest, exhibiting increased G0 to G1 phase cells (p<0.001) and decreased S-phase population (p=0.004) (Fig. 4C).

## Discussion

Already known as the first cause of mortality in men, non-small cell lung cancer (NSCLC) is nowadays a major cause of cancer-related death in women. Lung adenocarcinoma is the most common histological subtype, and its incidence has risen sharply in women, especially surpassing squamous cell carcinoma 28. Despite advances in diagnosis and treatments, the overall 5-year survival rate remains dismal, especially when lung cancer is diagnosed at advanced stages 9, 29. Therefore, a deeper understanding of the molecular mechanisms underlying lung carcinogenesis could contribute to the development of novel strategies for prevention and therapy. In our patient cohort, a high TKT expression served as a marker of poor prognosis in patients with LUAD.

Warburg identified a particular metabolic pathway in carcinomas that was characterized by the anaerobic degradation of glucose even in the presence of an abundant oxygen supply (aerobic glycolysis) 30. One of the main differences between normal and cancer cells is the difference in glucose metabolism. Cancer cells experience increased oxidative stress and metabolic reprogramming, and increased glycolysis in metabolism is thought to be associated with cell proliferation and survival 31. Therefore, inhibition of enzymes in the pathway could result in more potent anti-tumor effects. Non-oxidative glucose metabolism through the PPP promotes tumor cell proliferation and was controlled by Thiamine-dependent transketolase enzyme reactions 32, 33, different strategies using either thiamine antagonists (OT) or thiamin deprivation 33 have been suggested to deplete thiamin from cancer cells 34. Thiamine supplementation may stimulate high survival rates, proliferation rates, and chemotherapy resistance in tumor cells 35-37, but other studies have demonstrated a beneficial role for thiamine in cancer 38, 39. Thiamine deficiency can occur in cancer patients and lead to serious diseases, including Wernicke's encephalopathy 40. Our results showed transketoase of thiamine family plays a pivotal role in carcinogenesis and OT significantly exhibits an inhibitory effect on cancer. More cautious approach would be advisable before recommending the combined use of thiamine with other drugs in patients with cancer 17, 27.

We noticed that TKT expression increased progressively in LUAD stages I through III, but in stage IV there was a strong decreased. This is the first time that the expression of TKT has been correlated with tumor staging and metastasis in LUAD. Although TKT expression correlated with local tumor progression and regional lymph node metastasis, TKT expression in primary tumors decreased when distant metastasis occurred. This is probably due to the insufficient sample size of stage IV. But a similar pattern was also found in colorectal cancer and pancreatic ductal adenocarcinoma 15, 20. The PPP and glycolysis are interlinked, which are mechanistically relevant for the dynamic regulation of migration versus proliferation 41. PPP, which supplies ribose-5-phosphate and NADPH for biosynthetic processes, is elevated in rapidly proliferating cells but suppressed under acute severe hypoxic stress, favoring glycolysis to protect cells against hypoxic damage. In stage IV, the decrease of TKT may be related to downregulation of pentose phosphate pathway (PPP) enzymes and a flux shift towards glycolysis, which are causatively involved in regulating “go or grow” cellular programs due to hypoxia. Apart from hypoxia, a variety of other parameters can regulate the dichotomous balance between proliferative versus migratory functional programs including extracellular matrix components, growth factors, microRNAs, and transcription factors 42-44. Furthermore, the protein complexes independently reported in the STRING PPI database validated our hypothesis by the similarity of interaction patterns (Fig. S2). Certainly, more experiments will be required to verify above assumptions in the future.

Three transketolase genes have been identified in the human genome to date: transketolase (TKT), transketolase-like 1 (TKTL1) and transketolase-like 2 (TKTL2). Some researchers have suggested TKTL1 and TKTL2 are functional transketolases and represent novel therapeutic targets for diabetes and cancer 20, 45-47. TKTL1 might not possess transketolase activity. The lower levels of TKTL1 were found in cancer tissues than adjutant in the TCGA database. Our analysis showed there is no sense but an increasing trend in LUAD clinical samples. In previous study of TKTL1 in lung and other cancers, patients with a high TKTL1 expression in the primary tumors exhibited a worse prognosis compared to those with a low expression 48-51. TKTL1 is thus one candidate marker for risk identification. However, a recent study of a large cohort of colorectal cancer patients with liver metastases found that TKTL1 may serve as a reverse prognostic significance 20. In addition, TKTL1 served as a marker of a better prognosis in patients over 65 years old and among those with TNM class M1, stage IV disease, or perivascular invasion had already been described in pancreatic ductal adenocarcinoma 15. In lung adenocarcinoma disease, it remains unclear if a high expression alone indicates a better prognosis or if it reflects other invasiveness-reducing characteristics that we have yet to identify.

In conclusion, our study confirms previous findings that lung cancer patients with high TKT expression have a poor prognosis and highlight the importance of TKT as a potential therapeutic target. TKT inhibition may thus be a useful strategy to intervene in cancer cell invasion and metastases, and need to be explored a large cohort of clinical trial.

## Supplementary Material

Supplementary figures and table.

## Figures and Tables

**Table 1:**
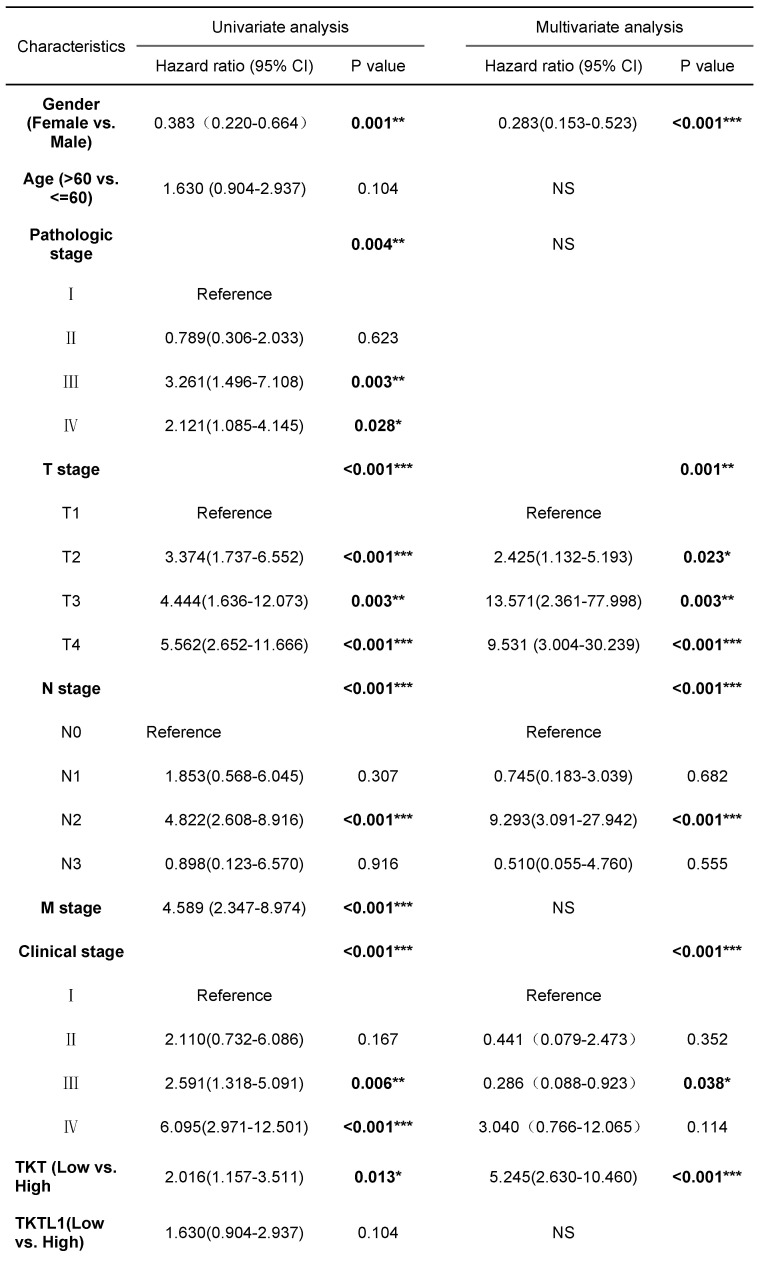
Univariate and multivariate Cox proportional hazards analysis of TKT expression and OS for patients with LUAD in the validation cohort

**Abbreviations: CI**, confidence interval; HR, hazard ratio. *, P<0.05; **, P<0.01; ***, P<0.001.
