# Supplementary material for: Transketolase Serves as a Biomarker for Poor Prognosis in Human Lung Adenocarcinoma
Source: J Cancer. 2022 May 13;13(8):2584–93. doi: 10.7150/jca.69583 (PMC9174852; doi:10.7150/jca.69583)
Supplement: Supplementary file 1 — Supplementary figures and table. [file jcav13p2584s1.pdf]

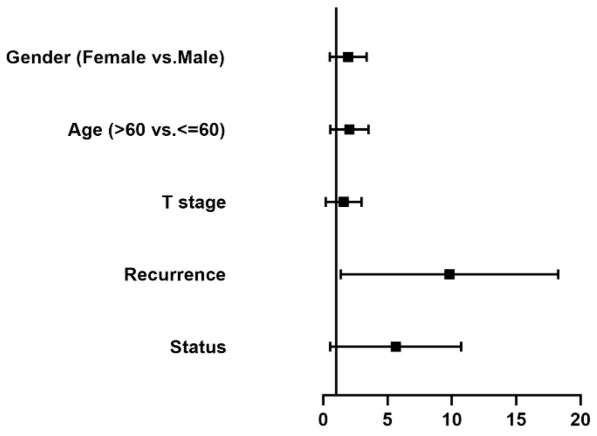

	Hazard ratio (95% CI)	P value
Gender (Female vs.Male)	1.583（0.715-3.507）	0.257
Age (>60 vs.<=60)	1.652（0.765-3.565）	0.201
T stage	1.162(0.431-3.129)	0.767
Recurrence	7.385(2.830-19.208)	0.001
Status	4.083(1.474-11.315)	0.007

Figure S1

Association between TKT expression and clinicopathologic features in stage Ⅰ patients in LUAD validation cohort

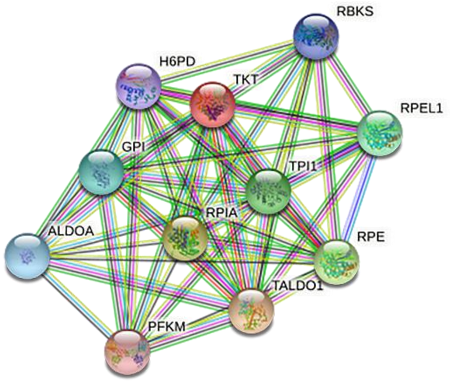

Figure S2 Interacting protein network of TKT was searched from String database.

Table S1. Univariate and multivariate Cox proportional hazard s analysis of TKT expression and DFS for patients with stage I LUAD in the validation cohort

Characteristics	Univariate analysis		Multivariate analysis	
	Hazard ratio (95% CI)	P value	Hazard ratio (95% CI)	P value
**Gender (Female vs. Male)**	0.395(0.203-0.768)	**0.006****	NS	
**Age (>60 vs. <=60)**	2.356(1.103-5.028)	**0.027***	NS	
**Pathologic stage**	NS		NS	
**T stage**		0.059	NS	
Tis	Reference			
T1a	0.380（0.185-0.781）	**0.008****		
T1b	0.779(0.441-1.378)	0.392		
T1c	1.308(0.564-3.037)	0.532		
T2a	1.453(0.779-2.712)	0.240		
**TKT (Low vs High)**	5.081(2.216-11.649)	**<0.001*****	3.177(1.357-7.440)	**0.008****

Abbreviations: CI, confidence interval; HR, hazard ratio. *, P<0.05; **, P<0.01; ***, P<0.001.
